# Conversion Characteristics and Production Evaluation of Styrene/*o*-Xylene Mixtures Removed by DBD Pretreatment

**DOI:** 10.3390/ijerph120201334

**Published:** 2015-01-26

**Authors:** Liying Jiang, Runye Zhu, Yubo Mao, Jianmeng Chen, Liang Zhang

**Affiliations:** College of Biological and Environmental Engineering, Zhejiang University of Technology, Hangzhou 310032, China; E-Mails: zhurunye@zjut.edu.cn (R.Z.); maoyb1224@163.com (Y.M.); jchen@zjut.edu.cn (J.C.); zhangliang039@sina.com (L.Z.)

**Keywords:** non-thermal plasma, dielectric barrier discharge, mixed VOCs, removal efficiency, biodegradability

## Abstract

The combination of chemical oxidation methods with biotechnology to removal recalcitrant VOCs is a promising technology. In this paper, the aim was to identify the role of key process parameters and biodegradability of the degradation products using a dielectric barrier discharge (DBD) reactor, which provided the fundamental data to evaluate the possibilities of the combined system. Effects of various technologic parameters like initial concentration of mixtures, residence time and relative humidity on the decomposition and the degradation products were examined and discussed. It was found that the removal efficiency of mixed VOCs decreased with increasing initial concentration. The removal efficiency reached the maximum value as relative humidity was approximately 40%–60%. Increasing the residence time resulted in increasing the removal efficiency and the order of destruction efficiency of VOCs followed the order styrene > *o*-xylene. Compared with the single compounds, the removal efficiency of styrene and *o*-xylene in the mixtures of VOCs decreased significantly and *o*-xylene decreased more rapidly. The degradation products were analyzed by gas chromatography and gas chromatography-mass spectrometry, and the main compounds detected were O_3_, CO_x_ and benzene ring derivatives. The biodegradability of mixed VOCs was improved and the products had positive effect on biomass during plasma application, and furthermore typical results indicated that the biodegradability and biotoxicity of gaseous pollutant were quite depending on the specific input energy (SIE).

## 1. Introduction

A large number of volatile organic compounds (VOCs) are emitted from various industrial processes and with their toxicity that threatens human health, they contribute to severe environmental problems [1,2], thus they are tightly controlled in many countries. Styrene and *o*-xylene, as typical VOCs, widely coexist in a wide range of industrial production processes, such as pesticide, surface coating and paint manufacturing processes, especially in the coatings industry [3,4]. China is regarded as the second largest country for the manufacture of coatings, therefore, large amounts of styrene/*o*-xylene mixture have been frequently released into the atmosphere accompanying the production and usage of coatings. Indiscriminate exposure to styrene/*o*-xylene mixtures may cause dizziness, neurasthenia and an increased risk of cancer [3,4]. Moreover, they could be precursors for the formation of secondary aerosols and photochemical smog. Therefore, styrene/*o*-xylene mixture emissions need to be strictly controlled by developing suitable abatement methods. 

Some well-established purification methods, such as adsorption, incineration, combustion and photocatalysis have been widely used to control the gas pollution. However, these methods exhibit some drawbacks including low efficiencies and high energy consumption [5,6]. Biological methods, developed in the late 80s, have received great attention due to their convenient maintenance, low operating costs and lower likelihood of secondary pollution. Many researchers have reported biofiltration processing as an effective technology for the removal of soluble and biodegradable volatile organic compounds (VOCs) [7,8,9,10]. However, its application is strongly limited in the case of biopoisonous and less biodegradable VOCs, such as *o*-xylene, which as the most recalcitrant BTEX that has demonstrated the lowest biodegradability in biofilters [11,12]. A new attempt to solve these problems is to combine biological purification with chemical methods, such as ozonation or photooxidation, and the role of chemical method in the combined processes is as a pretreatment. Recently, several studies have indicated that ultraviolet (UV) irradiation could be effectively utilized as a pretreatment, since it converts some recalcitrant VOCs into several simple intermediates. However, the application of UV is restricted by the relative long residence time required, and the removal rate falls sharply with increasing initial concentrations [13,14]. In our group, an integrated biotrickling filter (BTF) system with a non-thermal plasma (NTP) as the main pretreatment step was used, and the performance of the non-thermal plasma oxidation pretreatment step was examined. NTP, an emerging technology, makes the most of the supplied energy for creating energetic electrons, strong oxidizing agents and highly reactive species such as ozone, atomic oxygen O (O·), hydroxyl radical (HO·) [15,16]. Due to the presence of the above substances, it can achieve complete VOC oxidation to CO_2_ and H_2_O or convert them into non bio-poisonous and easily biodegradable byproducts, facilitating the subsequent biodegradation.

In real life, most industrial emissions contain VOC mixtures. However, the studied pollutants have often been single component VOCs [17,18,19,20]. Mok* et al.* studied the abatement of trichloromethane using nonthermal plasma reactors [21]. Zhang* et al.* compared styrene removal in air by positive and negative DC corona discharges, and reported that positive corona processing was more effective [20]. In the present study, a recalcitrant VOC mixture, styrene and *o*-xylene, was employed as the target contamination with a cylindrical DBD plasma configuration at room temperature. In order to identify the influence of key process parameters on the removal efficiency of styrene/*o*-xylene mixture to further achieve cost-effective process conditions, the influence of various parameters like initial concentration of the mixture, residence time and relative humidity in the plasma reactor were investigated, and the results are discussed. In addition, biodegradability and biotoxicity of byproduct(s) were also evaluated with the aim of providing fundamental data to evaluate the possibility of success of the subsequent biodegradation.

## 2. Experimental Section 

### 2.1. Experimental Set-Up

**Figure 1:**
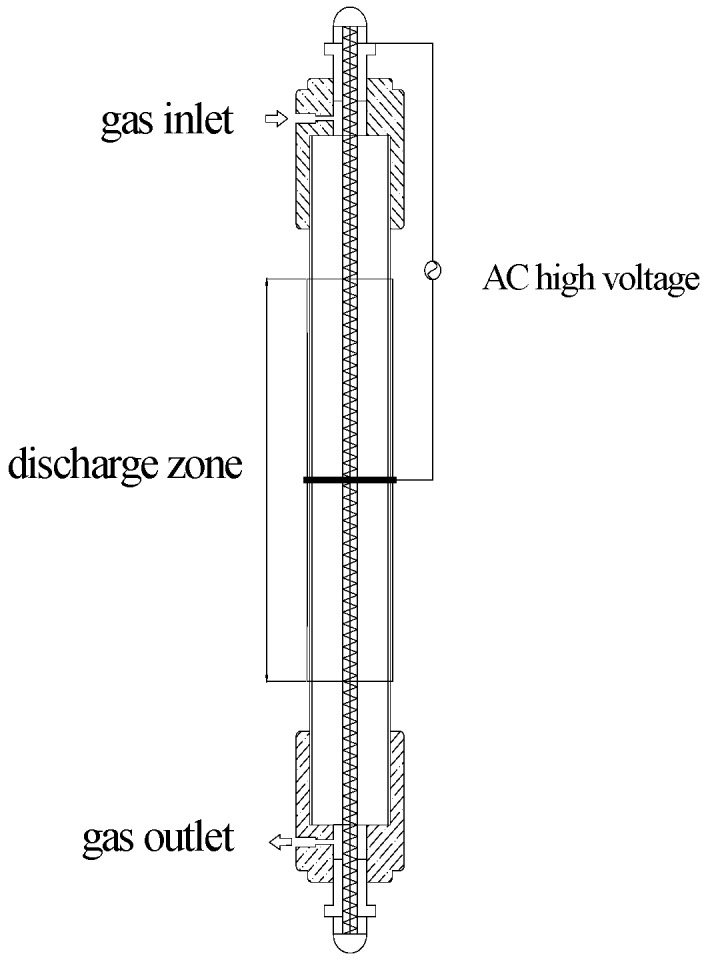
Schematic of the DBD plasma reactor.

The VOC decomposition performances were tested in a DBD reactor (Figure 1), including a cylindrical quartz tube as the dielectric barrier with an inner diameter of 18 mm and a coaxial 6-mm-thick stainless steel screw served as inner electrode, which was connected to an AC high voltage source.

Copper wrapping on the outer tube acted as the ground electrode. The discharge length was fixed at 20 cm and discharge gap between inner diameter and quartz tube was 6 mm. The applied voltage with the frequency of 10 kHz was varied in the range 4–9 kV and measured by a 1000:1 high voltage probe (Tektronix P6015A, Portland, OR, USA). Specific input energy (SIE) of the discharge was defined as:

(1) $$SIE(J/L)=\frac{Power(W)}{Gas\;flow\;rate(L/s)}$$

**Figure 2:**
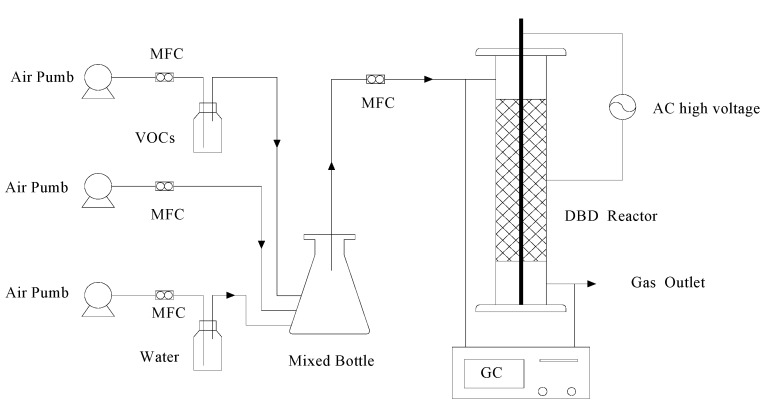
Schematic of the experimental setup.

The schematic diagram of the experimental setup was shown in Figure 2, consisting mainly of gas supplying system, DBD reactive system and product analytical systems. The mixture of VOCs was evaporated by bubbling with compressed air, then passed though the gas-mixer and diluted with additional air to a set concentration. Finally, the diluted VOCs were fed into the plasma reactor regulated by mass flow controllers with a Teflon tube.

### 2.2. Experimental Procedure

Experiments were carried out by changing the flow rates of streams to achieve different initial concentrations, residence times and relative humidity. The performance was evaluated by the removal efficiency of VOCs and CO_x_ selectivity, which are defined by the following relations:

(2) $$\eta (\%)=\frac{{\left[mixture\right]}_{in}-{\left[mixture\right]}_{out}}{{\left[mixture\right]}_{in}}\times 100$$

(3) $$Sco_{2}(\%)=\frac{\left[CO_{2}\right]}{8({\left[C_{8}H_{8}\right]}_{in}-{\left[C_{8}H_{8}\right]}_{out})+8({\left[C_{8}H_{10}\right]}_{in}-{\left[C_{8}H_{10}\right]}_{out})}\times 100$$

(4) $$Sco_{x}(\%)=\frac{\left[CO_{x}\right]}{8({\left[C_{8}H_{8}\right]}_{in}-{\left[C_{8}H_{8}\right]}_{out})+8({\left[C_{8}H_{10}\right]}_{in}-{\left[C_{8}H_{10}\right]}_{out})}\times 100$$

where [mixture]_in_ is the concentration of styrene and *o*-xylene introduced in the DBD reactor and [mixture]_out_ is the concentration of styrene and *o*-xylene in the effluent gas. [COx] is the sum of the outlet of concentration of CO and CO_2_.

### 2.3. Analytical Methods

The concentrations of *o*-xylene and styrene were measured with a gas chromatograph (Agilent GC6890, Palo Alto, CA, USA) equipped with a flame ionization detector (FID) [22]. The gas samples were taken using a six-way sampling wave and then transferred into a HP-Innowax capillary column (30 mm length, 0.32 mm diameter, and 0.5 μm film thickness). The FID temperatures was constant at 200 °C, and oven temperature was held fixed at 90 °C for 4 min , increased to 150 °C at the rate of 30 °C·min^−1^, and then held constant for 0.5 min. Whereas the relative humidity of mixed VOCs in the inlet was determined with humidometer (Testo 625, Lenzkirch, Germany).

The final products CO and CO_2_ were simultaneously measured using a gas chromatograph (GC6890, Agilent) equipped with a thermal conductivity detector (TCD) [3]. The oil-like byproducts were dissolved in acetone and further detected by gas chromatography and mass spectrometry (GC-MS7980, Agilent) [3]. The concentration of O_3_ was measured by a iodometry method [1].

For the purpose of achieving the best treatment effect with the subsequent BTF biological purification technology, a study on the biotoxicity of any byproducts is a prerequisite. The off-gas from each sampling condition was sparged into a 250 mL Erlenmeyer flask filled with 200 mL phosphate buffer for 30 min. The samples were added with *Chlorella* of regular growth and nutrient solution followed by artificial culture. The final biotoxicity results were expressed as biomass, which was calculated by measuring the OD_600_ of the absorption liquid every 12 h.

The BOD_5_/COD (B/C) method has been regarded as the most effective evaluation index to evaluate the biodegradability of organic compounds [23]. In general, the evaluation of the critical point was 0.3. When the ratio is greater than 0.3, the biodegradability of organic compounds is considered better, otherwise is poor [24]. COD and BOD_5_ were detected by a rapid digestion spectrophotometry method and BOD automatic analyzer (WTW OxiTOP IS12, Munich, Germany), respectively.

## 3. Results and Discussion

### 3.1. Influence of the Initial Concentration on the Decomposition of the Mixture of VOCs

**Figure 3:**
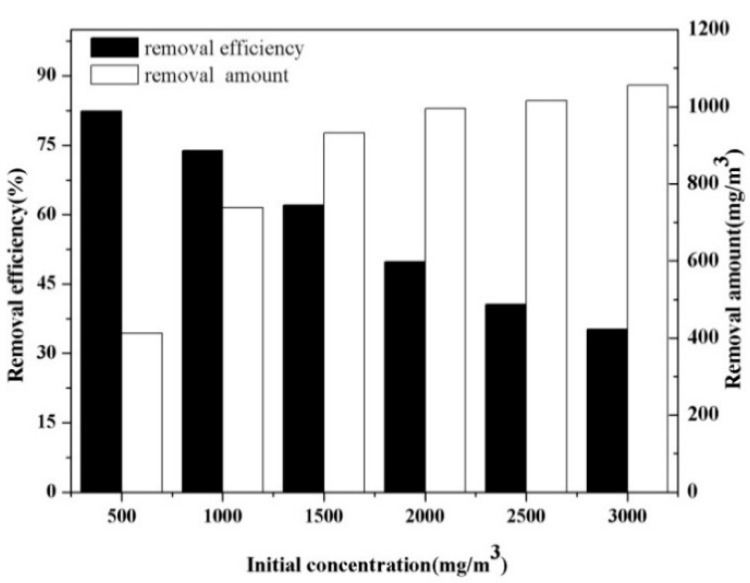
Influence of initial concentration on the decomposition of mixed VOCs.

Influence of VOC concentration on the decomposition of VOC was tested at a SIE of 400 J/L and a fixed residence time of 3.6 s as a function of the mixture of *o*-xylene and styrene concentration in the range of 500–3000 mg/m^3^ and the results are shown in Figure 3. 

An interesting observation from Figure 3 is that the removal efficiency decreased with the increasing concentration of the mixture of *o*-xylene and styrene, that is, the behavior of mixed VOCs decomposition strongly depending on the initial concentration. This observation was consistent with the findings of Gandhi *et al*. [4]. It has been observed that the removal efficiency was 82.4% for the initial concentration of 500 mg/m^3^, which decreased significantly to 35.2% when the concentration was increased to 3000 mg/m^3^. This can be explained by the fact that equal numbers of reactive species were generated by plasma with a constant input energy, when the initial concentration increases, each molecule shares less reactive species and electrons [18]. In other words, the input energy should correspondingly be raised to counterbalance the increased initial concentration. The diagram also shows another conclusion: despite the fact the removal efficiency decreased by degrees, the amounts of VOCs removed were rising until a balance was achieved. The removal amount for treating 500 mg/m^3^ VOCs was 412 mg/m^3^, and increased to 996 mg/m^3 ^for 2000 mg/m^3^. A small increase in the initial concentration of VOCs generally leads to an enhanced number of pollutant molecules introduced into the reaction area per unit time. Therefore, a higher collision probability of VOCs with reactive species is obtained, which finally leads to an increase in the removal amount.

### 3.2. Influence of Residence Time on the Decomposition of the Mixture of VOCs

**Figure 4:**
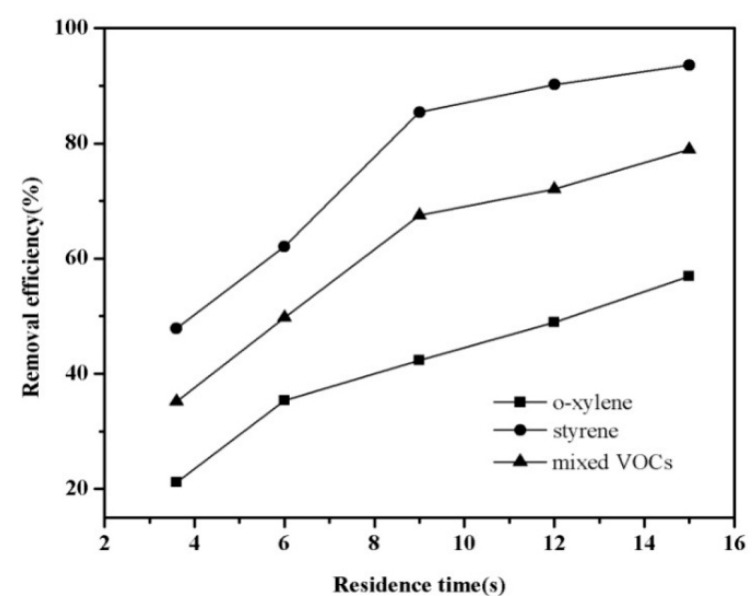
Influence of residence time on the decomposition of mixed VOCs.

In order to investigate the effect of residence time on removal efficiency, the residence time was varied between 3.6 to 15 s. These study was carried out with a fixed SIE, and inlet concentration of total VOCs was 3000 mg/m^3 ^(styrene = 1500 mg/m^3^, *o*-xylene = 1500 mg/m^3^) containing relative humidity of 50%–60%. Figure 4 shows the mixture of *o*-xylene and styrene abatement in the DBD reactor as a function of the residence time.

As expected, the VOC abatement was enhanced as the residence time of all the gases increased. As seen in Figure 4, the removal efficiency of styrene in the mixture was greatly increased from 47.9% at 3.6 s to 93.6% at 15 s, as well as that of *o*-xylene, whose removal efficiency increased from 21.2% to 56.9%. This phenomenon is attributed to extended interaction of gas pollutants and active radicals [25,26,27]. As shown in Figure 4, the removal of mixed VOCs increased rapidly as the residence time increased from 3.6 s to 9 s, and then the increase rate slowed down relatively as the residence time increased from 9 s to 15 s. Prolonging the residence time could further improve the removal efficiency of gas pollutants, but economical efficiency also should be taken into consideration. An overly high reaction time would lead to decreased energy efficiency due to the insufficient utilization of the short-lived reactive species [28]. 

Figure 4 illustrates that styrene was easier to decompose in the mixture, the the removal efficiency of the VOCs followed the trend styrene > the mixture of styrene and *o*-xylene > *o*-xylene. The abatement of styrene, the mixture and *o*-xylene operating at 9 s were 85.4%, 67.5% and 42.3%, respectively. The result could be explained by the competition of each component VOC molecule and active radicals. Styrene was superior to *o*-xylene with respect to the interaction with highly reactive species. In addition, previous studies demonstrated that chemical bond strength of VOCs and their molecular stability play an important role in the destruction of gas pollutants [29,30,31]. Among the mixed VOCs in this work, the bond energy of carbon-hydrogen bond in *o*-xylene is stronger than that of the carbon-carbon double bond in styrene, and therefore styrene can be more easily broken by energetic electrons.

### 3.3. Influence of Relative Humidity on the Decomposition of the Mixture of VOCs

**Figure 5:**
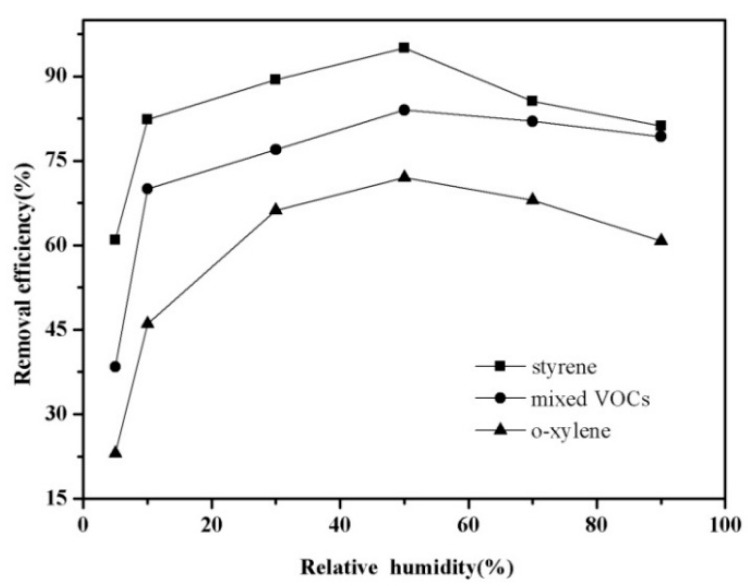
Influence of relative humidity on the decomposition of mixed VOCs.

The influence of humidity on the decomposition of VOCs has been studied for several VOCs [15,32]. Water vapor could enhance the removal efficiency since it may evolve into hydroxyl radical (HO·), whose oxidation power is superior to other oxidants like O_3_ and oxygen atoms (O·). However, it also has been reported that an excess of water seemed to have a negative effect. Therefore, it is necessary to determine the optimal humidity in order to achieve the best removal efficiency. Figure 5 presents the influence of humidity on the VOCs abatement at constant SIE of 400 J/L during the destruction of 3000 mg/m^3 ^mixed VOCs.

Typical results indicated that the removal efficiency of mixed gas rose at first as the humidity increased, and then decreased with an excess increase in humidity. For the example of *o*-xylene, it showed 23% removal at relative humidity of 10%, that improved up to 72.0% under 50% humidity conditions, followed by a decrease to 60.7% at 90% humidification, which illustrated the optimum humidity range was 40%–60%. The same results could be observed for styrene and total mixed VOCs. Therefore, adding small amounts of water vapor appeared to be more favorable from the point of view of VOC decomposition. Such an interesting phenomenon is the result of several factors. The initial enhanced removal efficiency may due to the formation by H_2_O ionization of hydroxyl radical, which is a strong oxidant can oxide the gas pollutants efficiently [33]. Meanwhile, the presence of water vapor also has a negative effect on decomposition of target pollutant that can be mainly ascribed to its electronegativity characteristics which limit the electron density and quench activated chemical species [34,35].

### 3.4. Interaction of Each Gaseous Compound in the Mixed VOCs

**Figure 6:**
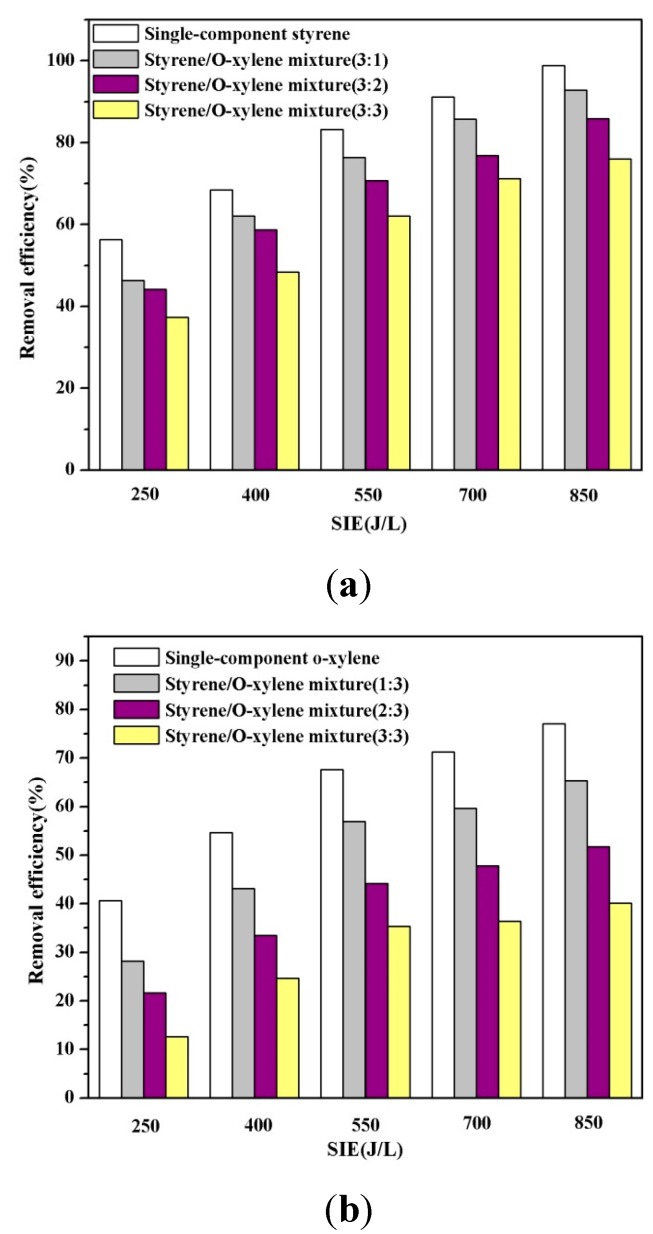
(**a**) Comparison of styrene removal efficiency for single-component and mixed VOCs; (**b**) Comparison of *o*-xylene removal efficiency for single-component and mixed VOCs.

The interaction of each gaseous compound in the mixed VOCs was explored by controlling the relative humidity within the range of 40%–60%. The residence time of simulated gas was constant at 6 s for the DBD reactor and with the different ratios of styrene/*o*-xylene mixture concentrations. Figure 6 shows the relationship between removal efficiency and SIE.

By comparing the removal efficiency of each VOC in the mixture, it was shown that styrene was the compound that was more easily degraded, and similar observation was made for each single-component VOC. At the same time, compared with the removal of single-component VOCs, a great decrease was observed in the removal efficiency of styrene and *o*-xylene when they were mixed together, no matter the ratio. The mixed VOCs share some reactive species and electrons, resulting in the observed decrease of removal efficiency. When the ratio was 1:1, the maximum removal efficiencies of styrene and *o*-xylene in the mixture were decreased 22.9% and 37.0% compared to the single-component VOCs, respectively, which also demonstrated that *o*-xylene decreased more rapidly. Therefore, mixing with styrene had much more effect on the removal efficiencies of *o*-xylene. The main reason may be that it is easier for styrene to react with energetic electrons and reactive radicals than *o*-xylene under the same experimental conditions. When the styrene was introduced into the reaction system with *o*-xylene, styrene was degraded preferentially, so that the amount of *o*-xylene removal was reduced with the decreasing amount of reactive radicals that reacted with *o*-xylene. Furthermore, on the other hand, Wang* et al.* [31] have reported that the total energy efficiency was much larger than that of any single-compound when various VOCs were treated at the same time, meaning that treatment of mixed VOCs was favored to enhance the utilization efficiency of radicals or electrons.

### 3.5. Generation of CO_X_ duringthe Decomposition of the Mixture of VOCs

**Figure 7:**
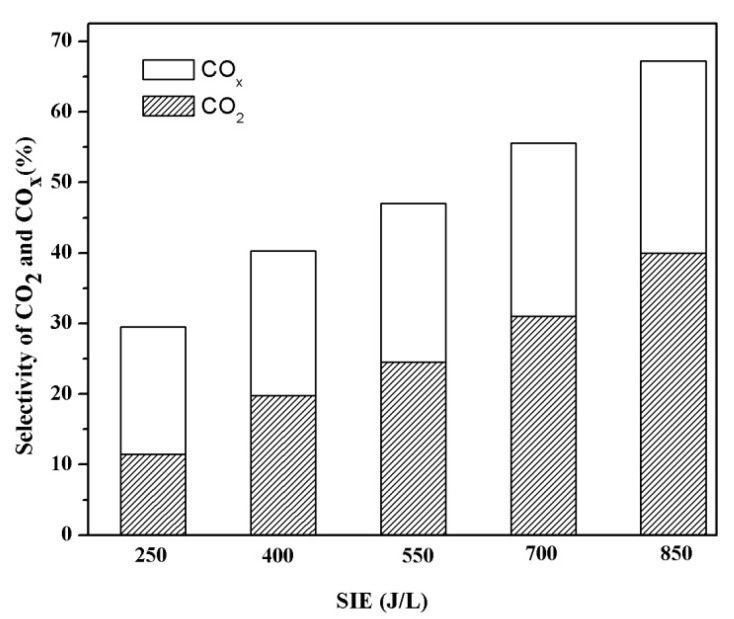
Selectivities of COx and CO_2_ as a function of the specific input energy.

The selectivity of gaseous products COx (CO + CO_2_) can directly reflect the extent of VOC mineralization. Therefore, the generation of COx during 3000 mg/m^3 ^styrene/*o*-xylene mixture degradation was measured, and the results are illustrated in Figure 7.

The results suggested that the selectivities of COx were enhanced with the increase of SIE. When the SIE was 250 J/L, the CO and CO_2_ selectivities were only 18.0% and 11.5%, respectively, and the remainder may be converted to gaseous intermediates or undesired solid carbon deposits on the surface of the electrode and inner walls of the reactor [3]. Meanwhile, the CO and CO_2_ selectivities reached 27.2% and 40% on increasing SIE to 850 J/L. The higher COx selectivity could be due to the oxidation of intermediates by more reactive species generated at higher SIE [36]. However, the SIE can’t be limitlessly prolonged because of the high cost. Therefore, the subsequent biological treatment is significant for the balance of handling expenses and extent of mineralization.

### 3.6. By-Products Analysis

**Figure 8:**
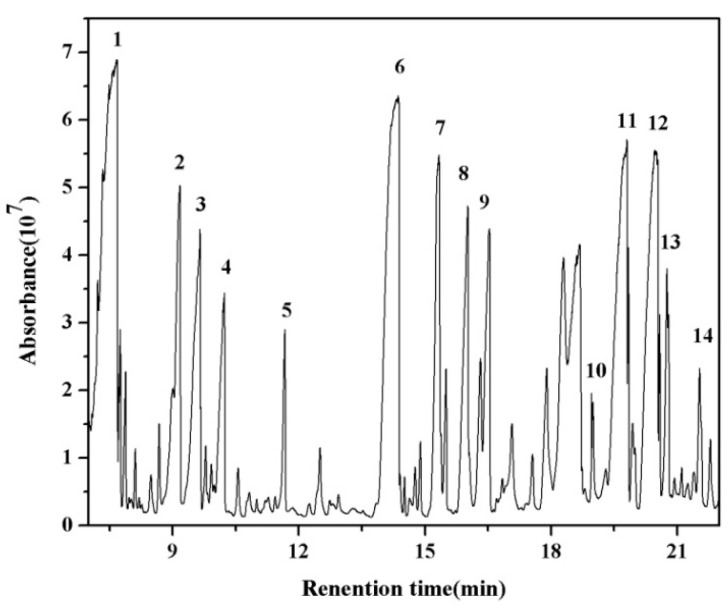
GC-MS chromatogram of intermediate-derivatives.

As seen from the results of CO_X _selectivity, it is easy to find that DBD technology caused incomplete mineralization, indicating the formation of large amounts of by-products. In this study, the GC-MS technique was used for the identification of byproducts, including air byproducts in the outlet and oil byproducts on the tube walls. The GC-MS chromatogram is shown in Figure 8 and some identified compounds are listed in Table 1.

**Table 1:** Main oil byproducts detected by GC-MS.

Peak No.	Byproducts	Peak No.	Byproducts
1	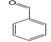	2	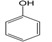
3	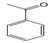	4	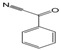
5	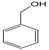	6	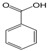
7	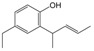	8	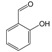
9	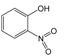	10	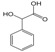
11	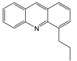	12	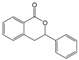
13	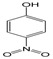	14	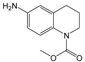

From Figure 8, about 14 kinds of main byproducts were detected in the DBD reactor at 250 J/L, among which some compounds with complicated structures, nitrogenous organics and benzene ring compounds were detected. The observation of nitrogenous organics indicated that the energy that generated in the reactor was higher than the dissociation energy of the nitrogen to nitrogen triple bond (9.8 eV). The reaction is mainly initiated via the collision between an electron in plasma and O_2_, H_2_O, N_2_ molecules with the formation of large number of radicals (O·, ·OH, N·). A phenyl radical and a benzyl radical generated during the collision between styrene/*o*-xylene molecules and an electron are trapped by radicals (O·, ·OH, N·), leading to the formation of aromatic compounds and nitrogenous organics [5]. 

**Scheme 1:**
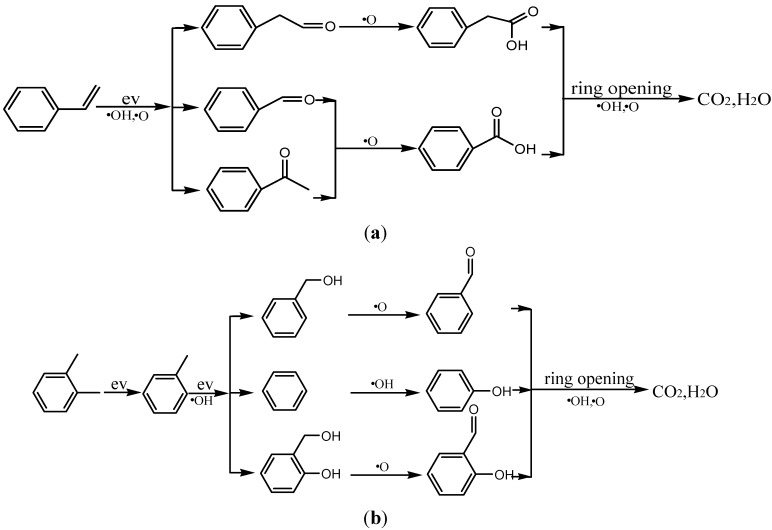
(**a**) Proposed reaction pathways for the abatement of styrene in gas phase; (**b**) Proposed reaction pathways for the abatement of *o*-xylene in gas phase.

According to the byproducts detected in the reactor, reaction pathways for the abatement of styrene and *o*-xylene in the gas phase are presented in Scheme 1a,b, respectively. The principle processes of the styrene/*o*-xylene mixture destruction induced by energetic electrons in plasma are electron and radical impact dissociation of molecules (O·, ·OH). The removal of styrene/*o*-xylene mixture depends on two removal mechanisms, including direct removal caused by the collision of electrons with styrene/*o*-xylene, and the reactions between styrene/*o*-xylene molecules and gas-phase radicals (O·, ·OH) [3,5].

### 3.7. Formation of O_3_ duringthe Decomposition of the Mixture of VOCs 

**Figure 9:**
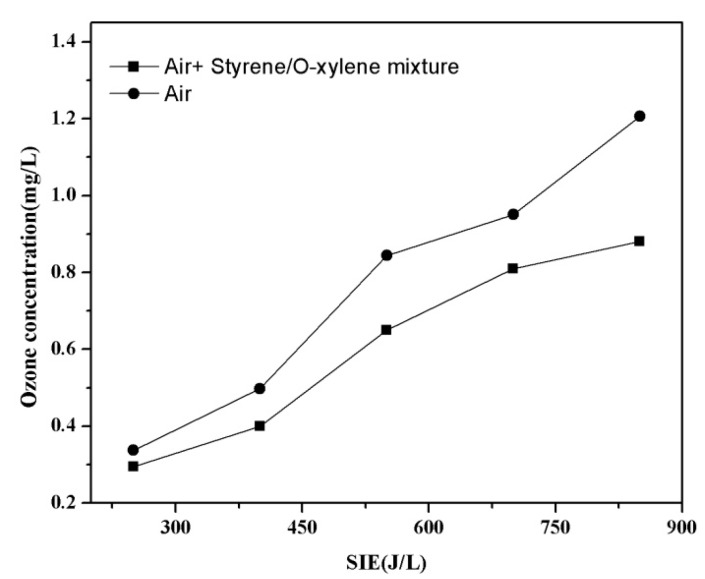
O_3_ concentration as a function of the specific input energy.

Ozone is customarily considered as an inevitable by-product produced by air plasma reactors. In air plasma, the ozone is formed through the reaction between an oxygen molecule and atomic oxygen which is produced by electron collisions on O_2_ [36]. The variations of the ozone concentration as a function of SIE are illustrated in Figure 9. In this set of experiments, the initial concentration of styrene/*o*-xylene mixture was kept constant at 3000 mg·m^−3 ^and the residence time was 6 s.

An interesting observation from Figure 9 is that the presence of styrene/*o*-xylene mixture caused a noticeable decrease in the concentration of O_3_. The O_3_ concentration was 0.84 mg/L at 550 J/L without VOC, and when styrene/*o*-xylene mixture was introduced into the reactor, the O_3_ concentration decreased to 0.65 mg/L. This phenomenon can mainly be explained by the inhibition of ozone generation because of the consumption of reactive species and energetic electrons during the styrene/*o*-xylene mixture degradation process [36]. Another reason is that O_3_ probably participated directly in the VOC oxidation, but at a relatively low rate because of the lower reaction rate between O_3_ and gaseous organic compounds [3].

### 3.8. Biodegradability of Byproduct

**Figure 10:**
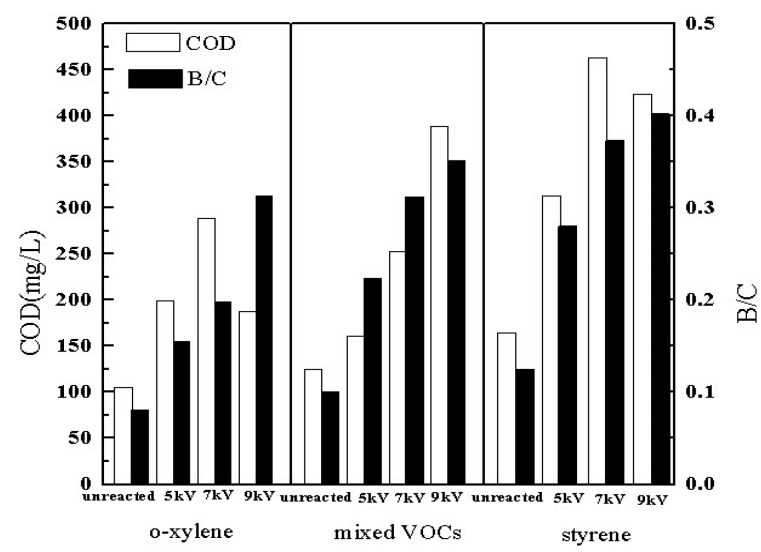
Biodegradability of byproducts.

The B/C values of single-compound styrene, singe-compound *o*-xylene and the mixture of styrene and *o*-xylene are presented in Figure 10 as a function of discharge voltage. The initial concentration was set at 3000 mg/m^3 ^and the relative humidity in the feed gas was 40%–60%.

As seen from Figure 10, the plasma reactor system enhanced the value of B/C of the single styrene, singe *o*-xylene and the mixed VOCs abatement compared to unreacted gases, respectively, meaning that the biodegradability of VOCs have been improved in the plasma reactor. To some extent, the COD of VOCs enhanced with the increasing discharge voltage, as well as B/C. As observed, the maximum value of B/C was achieved at the discharge voltage of 9 kV.

The higher biodegradability at a higher discharge voltage could be explained by an increased removal efficiency capable of producing more water-soluble and biodegradable byproducts. Styrene, *o*-xylene and styrene/*o*-xylene mixture achieved a value of B/C greater than 0.3 at 7 kV, 9 kV and 7 kV, respectively. Meanwhile, styrene and styrene/*o*-xylene mixture reached a maximum value of 0.4 and 0.35 at 9 kV, indicating that the byproducts of VOCs degraded by DBD pretreatment were susceptible to biodegradation, which was beneficial for the following biological technology processing.

### 3.9. Biotoxicity of Byproducts

**Figure 11:**
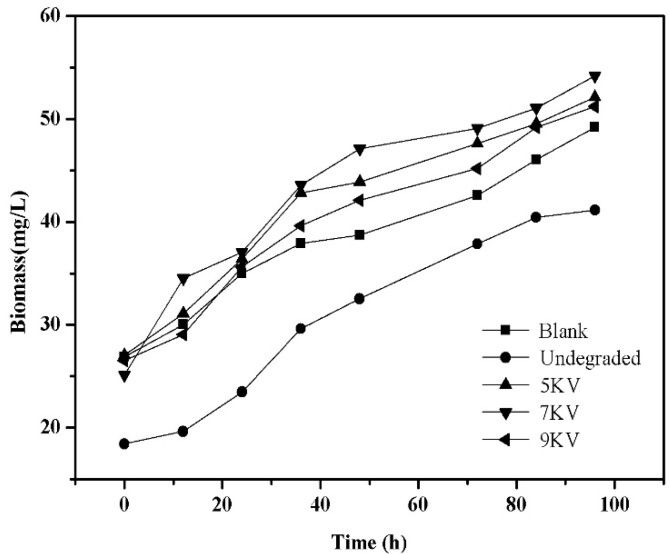
Influence of by-products on the growth of *Chlorella.*

The biotoxicity effect was investigated for different discharge voltage (5 kV, 7 kV, 9 kV) values and the relative humidity was fixed at 40%–60% with the gas mixture kept constant at 3000 mg/m^3^ (styrene = 1500 mg/m^3^, *o*-xylene = 1500 mg/m^3^). According to the previous results, the measurement of biotoxicity could depend on biomass, and the result is presented in Figure 11.

As shown in Figure 11, there was much difference in biomass among the undegraded gas and blank group. The undegraded gas has a negative effect on biomass, which indicated that styrene and *o*-xylene have strong biological toxicity for *Chlorella*. However, in case of plasma, the biomass showed different levels of increase. That is to say, the plasma pretreatment could transform recalcitrant VOCs into water-soluble and biodegradable by-products (alcohols, aldehydes, and acids) that can be utilized for biomass [37]. In addition, the biomass was much more dependent on applied voltage, giving values of 8.4 mg/L (7 kV)* versus* 5.1 mg/L (5 kV). This is reasonable because of the enhancement of mixed VOCs abatement in the presence of higher voltage, more water-soluble and biodegradable by-products (alcohols, aldehydes, and acids) were produced, which provide more carbon sources for biomass. In contrast, the biomass increment was lower at 9 kV compared to 7 kV, which could be attributed to the fact that high concentration of ozone and NOx produced by higher voltage can cause the inhibition of normal growth of *Chlorella* [36]. As seen from the Figure 9, the ozone concentration increased with the increasing discharge voltage, and reached a maximum at 9 kV in this study. Previous studies showed that moderate ozone had stimulative effect, but high concentrations of ozone are often regarded as a disinfectant that would kill microorganisms [38].

## 4. Conclusions 

The abatement of the mixture of styrene and *o*-xylene by non-thermal plasma generated in a DBD reactor was experimentally investigated under ambient temperature and pressure conditions. The experiment results of various technical parameters for mixed VOCs decomposition indicated that the removal efficiency was enhanced significantly with the rising reaction time, but overly high residence time is not favorable because of the low energy efficiency. Styrene was more easily degraded than *o*-xylene no matter whether as a single-component VOC or in mixed VOCs. The optimum relative humidity was 40%–60%, and the increase of initial concentration of VOCs had a negative effect on the removal efficiency of the plasma system. The removals of styrene and *o*-xylene decreased significantly when they were mixed together compared to those of the single-component VOCs, and *o*-xylene decreased more rapidly, indicating that the addition of styrene had an adverse on *o*-xylene removal. The selectivity of CO and CO_2_ was relatively low and much carbon deposition was observed on the surface of the reactor. Additionally, aromatic, nitrogenous by-products and O_3_ were detected in the reactor. It has been observed that the biodegradability of the mixture of styrene and *o*-xylene was strongly improved in the presence of NTP, which benefitted the subsequent degradation in a bio-trickling filter (BTF). During the research on the biotoxicity of VOCs, it was showed that the mixed VOCs in the absence of NTP has been transformed into water-soluble and biodegradable by-products that had a positive effect on biomass. Furthermore the positive influence of discharge voltage was remarkable. However, it is also necessary to avoid high voltages, which could inhibit the growth of *Chlorella* in terms of biomass due to the role of high voltage to produce high concentrations of ozone and NOx.
